# Cannabis and the Health and Performance of the Elite Athlete

**DOI:** 10.1097/JSM.0000000000000650

**Published:** 2018-08-28

**Authors:** Mark A. Ware, Dennis Jensen, Amy Barrette, Alan Vernec, Wayne Derman

**Affiliations:** * Alan Edwards Pain Management Unit, McGill University Health Centre, Montreal, Quebec, Canada;; † Department of Kinesiology and Physical Education, Faculty of Education, McGill University, Montreal, Quebec, Canada;; ‡ McGill Research Centre for Physical Activity and Health, Montreal, Quebec, Canada;; § Research Institute of the McGill University Health Centre, Translational Research in Respiratory Diseases Program, Montreal, Quebec, Canada.; ¶ Drakkar de Baie-Comeau, Quebec, Canada;; ‖ World Anti-Doping Agency, Montreal, Quebec, Canada;; ** Institute of Sports and Exercise Medicine, Surgical Sciences (Orthopedics), Faculty of Medicine and Health Sciences, Stellenbosch University, Cape Town, South Africa; and; †† IOC Research Centre, South Africa.

**Keywords:** cannabis, performance, pain, competition, doping

## Abstract

**Objective:**

Cannabis (marijuana) is undergoing extensive regulatory review in many global jurisdictions for medical and nonmedical access. Cannabis has potential impact on the health of athletes as well as on performance in both training and in competition. The aim of this general review is to identify and highlight the challenges in interpreting information with respect to elite athletic performance, and to point to important research areas that need to be addressed.

**Data Sources:**

A nonsystematic literature review was conducted using Medline and PubMed for articles related to cannabis/marijuana use and sports/athletic performance; abstracts were reviewed by lead author and key themes identified and explored.

**Main Results:**

Cannabis may be primarily inhaled or ingested orally for a range of medical and nonmedical reasons; evidence for efficacy is limited but promising for chronic pain management. Although evidence for serious harms from cannabis use on health of athletes is limited, one should be cognizant of the potential for abuse and mental health issues. Although the prevalence of cannabis use among elite athletes is not well-known, use is associated with certain high-risk sports. There is no evidence for cannabis use as a performance-enhancing drug.

**Conclusions:**

Medical and nonmedical cannabis use among athletes reflects changing societal and cultural norms and experiences. Although cannabis use is more prevalent in some athletes engaged in high-risk sports, there is no direct evidence of performance-enhancing effects in athletes. The potential beneficial effects of cannabis as part of a pain management protocol, including reducing concussion-related symptoms, deserve further attention.

## INTRODUCTION

The medical use of cannabis (“marijuana”) has largely been a patient-driven phenomenon because the illegal status of the plant *Cannabis sativa* and its constituents have hampered any meaningful drug development. However, interest in the therapeutic potential of compounds derived from cannabis (cannabinoids) has also been supported by recent scientific discoveries of the ubiquitous endogenous cannabinoid system (ECS) and its component receptors, ligands, and functional role in wide range of physiological processes. The effects of cannabis on athletic performance have recently been reviewed.^1^ This article will review this material, provide a broad context for the discussion, and highlight some novel considerations of cannabis use by athletes.

## BACKGROUND

It is commonly held that cannabis in crude extract or tincture form has been used for its analgesic properties throughout human history.^2^ Because the social and recreational use of cannabis began to become more widespread in the 1960s, the possibility that cannabinoids had potential therapeutic activity began to re-emerge. In 1964, a team of Israeli scientists published the structure of the primary psychoactive ingredient delta-9-tetrahydrocannabinol (THC)^3^ followed by the structure of another major (but nonpsychotropic) cannabinoid called cannabidiol (CBD).^4^ However the 1961, Single Convention on Narcotic Drugs had prohibited cannabis and its constituents, placing them in the highly restricted schedule IV. Despite some modest efforts to explore the therapeutic effects of THC for asthma,^5^ anxiety,^6^ and sleep,^7^ the potential therapeutic effects of cannabinoids largely disappeared from scientific view.

In the late 1980s, and into the 1990s, the medical use of cannabis became a political issue as patients with HIV/AIDS demanded access to the drug which they claimed helped with nausea, loss of appetite, and pain (Grinspoon, 1995 #5116); synthetic THC was approved for chemotherapy-induced nausea and vomiting (CINV) and anorexia associated with HIV/AIDS by the Food and Drug Administration (FDA) in 1992 (dronabinol; Marinol) and another synthetic THC derivative (nabilone; Cesamet) followed in 1995 with approval for CINV. In the meantime, in the United Kingdom, patients with multiple sclerosis were also politically active and demanded access to cannabis; this led to the development and launch of a plant-derived oromucosal spray (nabiximols; Sativex) in 2005 in Canada for the treatment of neuropathic pain and spasticity associated with multiple sclerosis and more recently for advanced pain associated with cancer.^8^ Furthermore, a preparation of cannabidiol (Epidiolex) has recently been shown to be effective in reduction of intractable seizures in children.^9,10^

## SCIENTIFIC AND CLINICAL EVIDENCE OF CANNABINOID ANALGESIA

Scientific interest in the analgesic effects of the cannabinoids has been fueled predominantly by increasing recognition of the components and function of the ECS, a ubiquitous family of G-protein–coupled cannabinoid receptors (CB1 and CB2) and lipid ligands (such as anandamide and 2-arachidonyl glycerol 2-AG). The ECS has been shown to play an important role in the modulation of a wide range of physiological processes, including neurotransmission, pain perception, and inflammation.^11^ It is not an exaggeration to state that all experimentation using animal models of pain in which the cannabinoid system has been targeted has suggested that harnessing this system has analgesic potential. Yet, the very entities that make the ECS an attractive therapeutic target also generate considerable challenges and somewhat undesired effects: activation of the CB1 receptor has widespread adverse effects on mood, movement, memory, and other processes that render it difficult to isolate the analgesic response from other behavioral effects. The characterization of the ECS and its components has led to interest in the development of drugs that target the ECS in an attempt to generate analgesia without such nonspecific effects: drugs that block endogenous ligand metabolic enzymes, drugs that inhibit ligand transport, drugs that bind only peripheral CB1 receptors and that do not cross the blood–brain barrier, and drugs that selectively bind the non-neuronal CB2 receptor have all been investigated in recent years. These pharmaceutical approaches have not yielded any strong leads to date.^12–14^

## CANNABIS USE AMONG ATHLETES

There is an apparent paradox in considering the effects of cannabis on athletic performance. Despite evidence that recreational cannabis use may acutely impair psychomotor skills and cognitive function, there is a perception among some athletes that cannabis use may have beneficial effects. The literature is scant, and the illegal or prohibited status of cannabis worldwide has limited our ability to generate high-quality data on the patterns and prevalence of cannabis use among elite athletes. In recent years, some attempts have been made to explore this phenomenon, and this section will review some of this published literature.

In interpreting data, it is important to note that studies of the prevalence of cannabis use among athletes may involve self-reporting or detection of cannabinoids after urine drug testing. Studies in which both methods were used have shown that under-reporting of cannabis use presents a significant risk of bias in self-report use studies.^15^ Cannabis is prohibited in sport (according to WADA) during the in-competition period only, which needs to be considered in the interpretation of self-reporting and antidoping analytic data.

Surveys of athletes have suggested that cannabis use is rare but may vary by sex and by sport.^16^ A recent systematic review by Brisola-Santos highlights some important observations.^17^ Self-reported cannabis use among NCAA athletes was predominantly for social or recreational purposes (61%); only 0.6% stated that the use of cannabis was primarily for performance-enhancing purposes.

There may be geographical associations as well, as cannabis use seems to be more prevalent in some countries compared with others; this may be associated with prevailing customs and attitudes.^17^ In several studies, the prevalence of cannabis use is second only to alcohol among athletes (in the general population, tobacco comes second). There is conflicting literature on the prevalence of cannabis use among elite and nonelite athletes, with some studies suggesting a higher prevalence of cannabis use among elite athletes and others suggesting the reverse; this variation may also relate to existing regional customs and perceptions regarding cannabis. Brisola-Santos et al suggest that “a number of athletic subgroups are at increased risk for marijuana use. Surprisingly, a common rationale for use seems to be to enhance sports performance”^17^ with particular associations with sliding sports (skeleton and bobsledding) and ice hockey.

## ATTITUDES TOWARD CANNABIS AMONG ATHLETES

Studies among amateur elite (NCAA) athletes suggest that the use of illicit drugs such as cannabis is mediated by social norms and risk of detection.^18^ This is important, as changing social attitudes and cannabis policies around the world may play an important role in changing use patterns of cannabis among athletes.^19,20^

Other behaviors reported to be associated with cannabis use include binge drinking and women competing at the international level. It also seems from one study that extreme athletes begin experimenting with cannabis at a very young age.^17^

Cannabis use among athletes may therefore be more related to social norms of behavior rather than to enhance performance.

## CANNABIS AND PERFORMANCE ENHANCEMENT

Among the first studies exploring the potential performance-enhancing effects of cannabis, Steadman and Singh (1975) exposed 20 healthy volunteers to smoke 1.4 g of cannabis containing 1.3% THC using a glass pipe; subjects took 20 to 25 puffs at each session. A placebo condition used cannabis with THC “chemically removed.” Subjects then underwent tests of muscular strength, physical work capacity, forced vital capacity (FVC), and flow rate of expiration. Cannabis use increased heart rate, systolic and diastolic blood pressure, and reduced physical work capacity. No change in hand grip strength, FVC, or expiratory flow rate was noted.^21^

In another early Canadian study, Renaud and Cormier exposed 12 young healthy volunteers to a single cigarette of smoked cannabis containing 1.7% THC and measured effects on FVC and FEV1, maximal work capacity (MWC). There was no placebo condition. Cannabis use reduced MWC compared with baseline, raised heart rate, and increased metabolic rate.^22^

As early as 1982, it was concluded that cannabis had no ergogenic potential and that “the dangers … far outweigh the advantage.”^23^ Eichner (1993) reported that cannabis was not ergogenic but rather ergolytic.^24^ Although specific performance-enhancing effects of cannabis may be in doubt, the use of cannabis to facilitate relaxation and reduce anxiety may be indirectly perceived to improve performance^25,26^ particularly in sports such as surfing and skiing. Athletes have also been shown to have higher pain thresholds than controls.^27^ Data on the effects of cannabis on quantitative sensory testing parameters are not conclusive but suggests that cannabinoids may increase punctate and pressure pain thresholds (Table 1). Cannabis use has also recently been identified as helping athlete's sleep time and recovery, which may favor performance when an athlete is facing multiple competitions in a short period.^28^

**TABLE 1.**
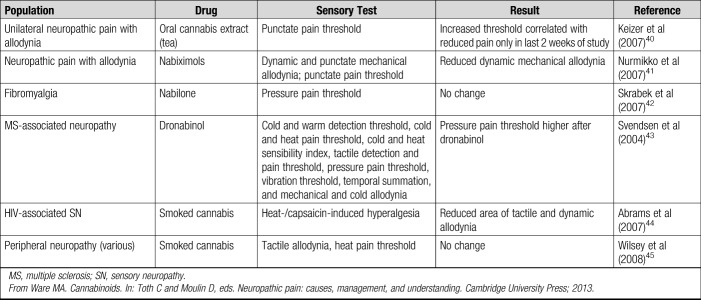
Effects of Cannabinoids on Quantitative Sensory Testing From Clinical Trials in Humans

It should be noted that anxiolytic and sedative medications as well as most pain medications are not prohibited. A lack of performance-enhancing effect has been echoed in more recent reviews,^29,30^ including the most recent systematic review,^1^ and this conclusion suggests that the reason for considering cannabis as a banned substance is due to safety concerns (see below) and the fact that use of an illicit substance is contrary to the spirit of sport.^31,32^

## CANNABIS AND SYMPTOM MANAGEMENT

In recent years, there has also been considerable demand from elite athletes for reconsideration of cannabis for its role as an analgesic and also for its role in reducing symptoms associated with traumatic brain injury. This has led, in particular, to calls from the National Football League Players' Association for reconsideration of their cannabis policy and for access to cannabis for medical purposes (http://gridironcannabis.org/). This public perception has brought the discussion back to the boardrooms of the governing bodies of professional sports, the International Olympic Committee and the World Anti-Doping Agency, and questions regarding the performance-enhancing effects of cannabinoids and their health effects on athletes (including athletes with impairments and Paralympians) have come back to the fore.

## CANNABIS AND THE PROHIBITED LIST

The List of Prohibited Substances and Methods (List) is published yearly by WADA, and substances are considered for inclusion if they meet 2 the following 3 criteria: (1) potential to or enhances sport performance; (2) actual or potential risk to health; and (3) against the spirit of sport. Cannabis has been on the WADA List since 2004. Despite the lack of hard evidence on the ergogenic effect, it is recognized that there are some health risks associated with the use of cannabis and many still believe that cannabis contravenes the spirit of sport, as defined in the World Anti-Doping Code.

Cannabis is prohibited in competition only. In recognition that a number of athletes were being sanctioned due to the fact that cannabis remained in their system after out of competition recreational use, the threshold level for cannabis metabolite carboxy-THC was raised in 2011 from 15 ng/mL to 150 ng/mL. In 2018, CBD was excluded from the List as it is not a cannabimimetic and does not have psychoactive properties.

Athletes may apply for a Therapeutic Use Exemption (TUE) to use a prohibited substance. These may be granted by a TUE Committee (TUEC) if the athlete would experience a significant impairment to health if the substance was withheld; the use would not enhance performance beyond a return to normal health; and there is no reasonable nonprohibited alternative. These criteria are judged on a case by case basis and subject to interpretation by TUECs.

## CONSIDERATION OF CANNABIS USE IN ATHLETES WITH IMPAIRMENTS

The reported epidemiology of injuries in Paralympic athletes indicates that many of them may have pain, either form the injury itself or as a result of the impairment (see accompanying article by Grobler et al in this edition). Of the eligible impairment types, several of them (including postamputation pain and central neuropathic pain related to spinal cord injury) may theoretically benefit from THC use^33^ and nabilone,^34^ and patients report improvement in spasticity^35^ and therefore might be valuable in the management in athletes with brain injury with resultant spasm and pain. In addition, glaucoma, a leading cause of visual impairment, may also be cannabinoid responsive as cannabinoids such as THC reduce intraocular pressure, but clinical data are lacking.^36^ Although a TUE can be granted to athletes with impairment who have been prescribed medical cannabis, no specific clinical trials have been conducted in this population of athletes.

## SAFETY CONSIDERATIONS

The possible adverse health effects of cannabis in elite athletes have not been specifically addressed, but lessons may be drawn from other recreational cannabis literature. In a recent review,^37^ cannabis adverse effects were considered as acute or chronic. Acute effects include effects on memory, coordination, and judgment (and paranoia/psychosis after high doses). Chronic effects are largely associated with early adolescent cannabis use and include dependence, poor school performance, and altered brain development; other effects include chronic bronchitis (from inhalation) and increase in risk of chronic psychosis disorders in those with a predisposition to such disorders.

Most of the work reviewed to date on cannabis effects on performance or safety have focused on recreational use rather than specifically authorized medical use; little is known of the adverse effects of cannabis when used under medical supervision, however, effects seem modest and well-tolerated.^38,39^

## CONCLUSIONS

Cannabis use among athletes may reflect external societal and cultural norms and experiences within certain subcultures of sport. Cannabis use is more prevalent among some athletes engaged in high-risk sports, but there is no evidence of performance-enhancing or causal effects. Self-reported use of cannabis for pain and concussion management among elite athletes is increasingly being reported, and with emerging scientific appreciation of the potential physiological role of the endocannabinoid system deserves serious further inquiry. Examples of specific research questions that require attention are the use of cannabinoids to reduce opioid pain medication and the possible role of cannabinoids to prevent or manage symptoms of traumatic brain injury.
